# Social exclusion and universal health coverage: health care rights and citizen-led accountability in Guatemala and Peru

**DOI:** 10.1186/s12939-020-01308-y

**Published:** 2020-12-09

**Authors:** Jeannie Samuel, Walter Flores, Ariel Frisancho

**Affiliations:** 1 Health and Society Program, York University, Toronto, Canada; 2 Center for the Study of Equity and Governance in Health Systems (CEGSS), Guatemala, Guatemala; 3 CMMB Peru, Lima, Peru; 4 ForoSalud Peru, Lima, Peru

## Abstract

**Background:**

While equity is a central concern in promoting Universal Health Coverage (UHC), the impact of social exclusion on equity in UHC remains underexplored. This paper examines challenges faced by socially excluded populations, with an emphasis on Indigenous peoples, to receive UHC in Latin America. We argue that social exclusion can have negative effects on health systems and can undermine progress towards UHC. We examine two case studies, one in Guatemala and one in Peru, involving citizen-led accountability initiatives that aim to identify and address problems with health care services for socially excluded groups. The case studies reveal how social exclusion can affect equity in UHC.

**Methods:**

In-depth analysis was conducted of all peer reviewed articles published between 2015 and 2019 on the two cases (11 in total), and two non-peer reviewed reports published over the same period. In addition, two of the three authors contributed their first-hand knowledge gathered through practitioner involvement with the citizen-led initiatives examined in the two cases. The analysis sought to identify and compare challenges faced by socially excluded Indigenous populations to receive UHC in the two cases.

**Results:**

Citizen-led accountability initiatives in Guatemala and Peru reveal very similar patterns of serious deficiencies that undermine efforts towards the realization of Universal Health Coverage in both countries. In each case, the socially excluded populations are served by a dysfunctional publicly provided health system marked by gaps and often invisible barriers. The cases suggest that, while funding and social rights to coverage have expanded, marginalized populations in Guatemala and Peru still do not receive either the health care services or the protection against financial hardship promised by health systems in each country. In both cases, the dysfunctional character of the system remains in place, undermining progress towards UHC.

**Conclusions:**

We conclude that efforts to promote UHC cannot stop at increasing health systems financing. In addition, these efforts need to contend with the deeper challenges of democratizing state institutions, including health systems, involved in marginalizing and excluding certain population groups. This includes stronger accountability systems within public institutions. More inclusive accountability mechanisms are an important step in promoting equitable progress towards UHC.

## Introduction

Universal health coverage (UHC) has quickly risen in the global health agenda in recent years. Since the WHO advanced the case for UHC in its World Health Report on health system financing in 2010, there has been a great upsurge of demand for advice and assistance about how to deliver UHC [1], and a growing number of countries “across the development spectrum” have been working towards UHC [2]. UHC has been endorsed by the World Bank and integrated into the UN Sustainable Development Goals (SDG) as a specific target for all countries (target 3.8 achieve UHC by 2030)—identifying it as both a global health and a development priority [3]. A 2019 report by the Commission of the Pan American Health Organization on Equity and Health Inequalities in the Americas also endorses the push toward UHC, arguing that “universal access to health care should be a feature of all societies” [4].

The idea of equity is essential to Universal Health Coverage. UHC is intended to make quality health services available and accessible to all. However, we argue that scholarship on UHC does not take social exclusion into sufficient account in its thinking on equity. The impact of social exclusion on equity in UHC remains underexplored. This is problematic since there are important insights to be gained from understanding the challenges faced by socially excluded groups for achieving UHC. In particular, we argue that social exclusion can have negative effects on the design, funding and quality of health care services in ways that can undermine efforts to extend UHC. To demonstrate this, we examine two case studies, one in Guatemala and the other in Peru, where grassroots volunteers from Indigenous communities are involved in citizen-led accountability initiatives in order to improve community access to quality health care services. These case studies help to illustrate the challenges faced by socially excluded groups to receive effective UHC in highly unequal countries such as Guatemala and Peru. We conclude that efforts to promote UHC cannot stop at increasing health systems financing. In addition, these efforts need to contend with the deeper challenges of democratizing states and health systems that are involved in marginalizing and excluding certain population groups.

### UHC and social exclusion

Universal health coverage (UHC) is achieved when all people receive quality health services that meet their needs without being exposed to financial hardship [5]. Conceptually, this involves strengthening all aspects of health systems including public health and population measures involving promotive and preventative services. However, the mainstream discussion on UHC places primary emphasis on the financing mechanisms related to the provision of health services, including the extension of health insurance [5]. As a result, to date, much of the literature seeks to inform decisions about how to allocate scarce resources, define priorities, and overcome barriers to extending coverage. Some studies discuss principles and obligations for equity [5, 6], while others review the experiences of countries that have expanded coverage in order to identify lessons learned as well as challenges and opportunities [7–11]. Much of this latter research in particular acknowledges the importance of political economy concerns to UHC reforms [9, 11–13]. Progress towards UHC requires redistribution and inevitably involves “political trade-offs, conflicts and negotiations” [9]. As Stuckler et al. observe, “Adopting UHC is primarily a political, rather than a technical issue” [11]. Similarly, how and how far equity is pursued in UHC will depend upon how political and economic concerns are navigated [4].

We argue that, despite the recognition of the role of politics in UHC, much of the discussion has not yet paid sufficient attention to social exclusion as an important factor influencing the realization of UHC. Social exclusion refers to a “set of structural mechanisms that prevent certain social groups from fully participating in the economic, social, political and cultural spheres of society” [14]. These mechanisms can limit access to health, housing, employment, education, political representation, citizenship, humane treatment, etc. [15]. Exclusion is a broad term and refers to people who are stigmatized and marginalized in many different ways such as racialized and ethnic minorities, drug users, persons with disabilities, sexual and gender minorities, migrants, refugees, and Indigenous peoples. People also face multiple forms of exclusion by falling into combinations of categories, such as gender and racialization. This leads to intersecting or compounding forms of discrimination. Social exclusion is related to other concepts such as structural violence and systemic racism in that it focuses on the structural in a context of unequal power relations [16]. As with these related concepts, exclusion is brought about by the interplay of social institutions, cultural distinctions, and political processes which create social boundaries that perpetuate inequality. Exclusion involves the creation and reproduction of stigmatized social categories, and “[d] isrespect, discrimination and degradation are as much at work as are monetary poverty and physical need” [17]. The concept is useful in that it directs attention to the multi-dimensional barriers to full participation in society and can help in the causal analysis of poverty and deprivation [18].

Health systems represent an institutionalized social setting where social divisions and patterns of exclusion can be combatted or reinforced [19]. The reproduction of social divisions is particularly visible where health systems have developed in a segmented fashion so that social groups are served by separate segments of the health care system. Health system segments that serve disadvantaged populations tend to provide lower amounts of funding per person, inferior quality of care, etc. [10, 20]. This form of social segregation can also normalize social divisions and make them more resistant to challenge [21].

Social exclusion raises obvious equity issues relevant to UHC, particularly in terms of who is covered and who is not. It can also have less visible effects on how health systems serve socially excluded populations. This is not unique to Latin America. Even where health coverage is extended, studies suggest that patterns of social exclusion can have important effects on the quality of services for disadvantaged groups. For example, extensive research has demonstrated that racialized and ethnic minorities in the U.S. receive lower quality of health services than white Americans, even after disease status, socioeconomic differences and other health care access related factors are taken into account [22, 23]. In addition, research in Canada and the U.S. shows that factors such as stereotyping and bias by medical practitioners contribute to persistent racial and ethnic disparities in health care [24–26].

In short, the literature on social exclusion in health care raises important questions relevant to the political economy and equity concerns involved in realizing UHC in Latin America. We argue that existing debates on UHC and equity would benefit from taking a more complete account of the consequences of social exclusion in health care.

### Citizen-led accountability initiatives as a window into social exclusion in health care

We use case studies concerning two citizen-led accountability initiatives in two Latin American countries to explore how social exclusion can negatively influence health coverage, and thereby inform broader debates about progress towards UHC. ‘Citizen-led accountability’ or ‘social accountability’ are terms used to describe initiatives involving grassroots groups that use new forms of civic engagement to promote reform in government [27, 28]. It is an evolving category that includes citizen monitoring of public services, social audits, and participatory budgeting [29]. Since the early 2000s, various citizen-led accountability initiatives have formed in countries around the globe to address problems with health care delivery for poor and neglected communities [30]. These initiatives are typically led by community volunteers who receive training from NGO allies to carry out various tasks in local health facilities. These include monitoring, documenting problems and issues, and advocacy [31]. These initiatives seek to promote change by building citizen power and civic engagement. They often depend on the establishment of participatory spaces in order to bring issues to the attention of local health officials [32].

We contend that these initiatives often serve as learning laboratories where both community volunteers and NGO allies make discoveries about the quality and delivery of local health services. Citizen-led accountability initiatives help to gather and focus collective knowledge concerning experiences that are often marginalized within mainstream policy narratives. Research on these kinds of initiatives provides additional insight into the relationship between social exclusion and health care services.

## Methods

This article is based on an in-depth analysis of all peer reviewed articles and book chapters published between 2015 and 2019 (11 in total) on two specific cases of citizen-led accountability in health care: one in Guatemala and the other in Peru [28, 30, 31, 33–41]. All but one of the peer reviewed studies [30] was co-authored by one or more of the authors of this present article. Through our analysis of these studies, we have sought to identify challenges faced by socially excluded populations to receive UHC. In particular, the case studies focus on social exclusion of Indigenous populations in relation to publicly provided health care services in both countries. Review of these studies was further supplemented with information from four published non-peer reviewed research reports concerning the cases [32, 35, 42, 43]. In addition, two of the three authors have contributed their personal knowledge gained through years of practitioner involvement with the citizen-led initiatives examined in the two case studies.

## Results

### Citizen-led accountability in Guatemala

Guatemala is one of the most unequal countries in the world, and it is a country where inequality is closely connected to ethnicity and race [44]. While overall poverty in the country remains persistently high, the rate of extreme poverty is three times higher among the Indigenous population than among the non-Indigenous population [44]. These inequalities are also reflected in health and nutrition outcomes. The rate of chronic malnutrition for Indigenous children under five in Guatemala is 58%, over twice as high as for the non-Indigenous child population [45]. Stark contemporary inequalities in health and income in Guatemala stem from a history of European colonization, decades of military dictatorships, the exclusion of poor and Indigenous populations from development, and a 30 year internal war [32]. In peace accords signed after the end of the civil war in 1996, the state made commitments to increase social investment for the most vulnerable. Despite modest increases in tax revenues and social spending since the 1990s, in 2015 Guatemala still had the third lowest domestic government health care expenditure per capita among countries in the Americas [4].

Poor communities, often in rural areas with large Indigenous populations, are served by a network of government health care facilities that are chronically underfunded and insufficient for the number of people they aim to serve. In the past two decades, different policy initiatives managed to expand basic health care service coverage. However, coverage has contracted in the past 5 years and funding has stagnated [46]. Meanwhile, a significant percentage of the country’s population remains in need of quality publicly provided health services. For example, a report from the Commission of the Pan American Health Organization on Equity and Health Inequalities in the Americas notes that 78% of female and 68% of male nonagricultural workers in Guatemala are employed informally [4]. These workers lack social protections from employers and depend on precarious income streams, thereby increasing the importance of publicly provided health services to meet their health service needs.

Beginning in 2007, a network of volunteer health defenders was catalyzed in Guatemala by CEGSS, an NGO focused on participatory action-research to reduce social exclusion and inequality in health care affecting the rural Indigenous population. Previously, CEGSS had sought to collect evidence itself of failing public services in Indigenous rural areas in order to press public officials for action. However, officials disregarded their message as politically motivated interference by outsiders [34]. CEGSS revised its strategy to provide capacity-building services to representatives elected from rural communities so that they could collect information on services and engage with officials themselves [35]. The passing of new laws, such as the Decentralization, Community Development Councils and Health Act, recognize the right and responsibility of citizens to participate in the planning, monitoring and evaluation of public services [32].

This led to the formation of the Network of Health Rights Defenders (REDC-SALUD in Spanish), a volunteer group of representatives elected by their rural communities to defend rights to health. The Network is now active in 30 municipalities in five provinces in Guatemala. CEGSS played an important support role in these organizing efforts, and it continues to provide training and resources to facilitate the network’s activities. Health defenders monitor local health facilities to assess the availability of essential drugs, medical supplies and health personnel as compared against national standards. They also monitor decision-making within health commissions and municipal governments. The information gathered through these activities is then used to press for accountability and change [32, 35, 42].

Monitoring carried out by members of the health defender network has revealed a series of serious problems compromising access to publicly provided health care services for rural populations, where many health users are Indigenous. Along with access issues, monitoring has also highlighted concerns about the low quality of health service provision in these areas [37]. Community health defenders involved in citizen-led accountability describe persistent issues with discrimination, abuse, cultural insensitivity and disrespectful treatment in local health facilities. This can involve a wide range of behaviours including yelling, forcing unwanted procedures on patients, giving priority to higher status individuals, refusing to seek out translation, or lying to patients and their families. In general, these acts serve to denigrate Indigenous health users, alienate patients from care, and reinforce a sense of their lack of belonging [33, 37].

Denial of service is another common problem identified by the health defenders in Guatemala. In many facilities, health providers stop intake by mid-morning, without regard for patients who have been waiting for hours, and contrary to national care standards and regulations. Monitoring also has revealed instances where patients are denied care for not speaking enough Spanish or not being ‘clean’ enough [33]. Without knowledge of the official rules governing health care providers, members of Indigenous communities are often not in a position to identify these actions as abuses.

Community health defenders also identified a common problem where patients are charged a fee for services or medications officially covered through the publicly provided health system. These charges are contrary to Guatemalan law which stipulates that all public services are free of charge at the point of delivery. Such fees may be charged for vaccinations, birth certificates or the use of a government ambulance during a medical emergency. Even when the sums being charged are relatively small, for the rural poor, these illegal, out of pocket expenses create a significant economic burden that public health coverage is designed to remove. These fees also erode the trust of socially excluded communities in their public health system and discourage health users from seeking out services [47].

Another problem flagged through monitoring concerns the frequent unavailability of medicines that are part of the government priority list scheme. Officially, these medicines are required to be available for free at government-run health facilities. However, families are regularly required to use their limited income or to borrow money to acquire prescribed medicines from private pharmacies [37].

Members of local communities were often unaware that local practices such as denials of service or illegal charges contravene Ministry of Health policies and regulations. Once communities learned of these rules, they increasingly reported violations to community health defenders [35]. Using various strategies, including meetings with local officials and complaints to the Human Rights Ombudsperson or the Attorney General’s office, the community health defender network has often been able to resolve issues at the local level. The network has helped to press certain health providers to change their behavior. However, successfully addressing issues that have structural causes has been much more challenging and underscores the embedded nature of social exclusion and inequalities.

Underfunding, mismanagement, corruption and neglect continue to cause significant problems for the publicly-provided health system in Guatemala. For example, media reports suggest that the problem with the availability of medicines appears to be in part due to procurement practices at the national level. It appears that political figures are able to redirect public procurement towards higher priced suppliers in exchange for kickbacks. The higher prices mean that fewer units are purchased which translates into shortages at the local level. Neither CEGSS nor the health defender network has been able to mobilize successfully against this problem to date [35].

### Citizen-led accountability in Peru

Peru is also a highly unequal country where persistent social and economic divides emerge from a colonial history, long periods of dictatorship and compromised democracy, and civil conflict [48]. Various groups, including Indigenous peoples and Afro-descendant populations, face persistent and multi-dimensional forms of social exclusion reflected in key social indicators. For example, the infant mortality rate among Indigenous peoples in Peru is 32 per 1000 live births, nearly triple the rate of 10 per 1000 for the country’s non-Indigenous population [4]**.**

Patterns of exclusion are reproduced in the country’s multi-tiered health system. People with the greatest access to financial resources are more likely to use private insurance and services, while salaried workers are covered by a separate system of facilities and services financed primarily through payroll taxes. Informal workers and the rural poor, many of whom are Indigenous, are left to rely on the least generous tier of the health system, where a network of health posts and basic health coverage are financed through public revenues. This includes a substantial segment of the population. For example, 2017 statistics from PAHO’s *Just Societies: Health Equity and Dignified Lives* report estimated that 67% of female nonagricultural workers and 52% of male nonagricultural workers were engaged in the informal labour sector, with precarious income streams and no access to employer benefits [4]. The system of health coverage for Peru’s poorest citizens was initially expanded in the 1990s, and received new investment with democratization and economic growth in the 2000s. In 2009, the government passed the Universal Health Insurance Law, which was intended to expand and deepen earlier provisions of health insurance by the state to marginalized populations, including the *Seguro Integral de Salud* (SIS). However despite these expansions, as of 2015, Peru’s domestic government health care expenditure per capita was still lower than at least 18 other countries in the Americas [4].

In 2007, a citizen monitoring initiative arose in Puno, a largely mountainous and predominantly Indigenous region of the country, partly as a result of a participatory study conducted in the mid 2000s by a US NGO, Physicians for Human Rights. The NGO was collecting stories regarding maternal deaths in the region. The study suggested that serious deficiencies in the health system were impeding access to proper maternal care and contributing to the exceptionally high maternal mortality rate in the region [49]. Once the study was complete, two Peruvian NGOs cooperating with PHR – CARE Peru and ForoSalud – began to recruit women from Indigenous communities in two districts in the region to participate in an effort to increase access and quality of care by holding local health care facilities accountable [31, 39, 43]. Those civil society partners helped to organize the initiative and provided training on participatory rights, health rights, and entitlements under the health and public insurance systems. They also were able to take advantage of a citizen participation law and Ministry of Health policies that recognize health rights in order to press health officials to accept a system of citizen monitoring of local health facilities in these districts. In addition, both the local Human Rights Ombuds office and the SIS regional office became partners in the initiative, contributing training and other forms of assistance to the volunteer monitors [31, 39, 43].

After receiving training, the citizen monitors carried out regular shifts at their local health facilities (health posts, health centres and local hospitals) where they observed practice, advised and advocated for patients, and documented problems. As Indigenous women, the monitors initially faced significant challenges carrying out their monitoring duties given the history of exclusion and difficulties with their local public health facilities that they and many of their community members had encountered over the years [31, 39, 40]. However, over time they collected information and complaints that they later shared with health officials in collective meetings with the monitors’ NGO and state allies [31, 36, 43, 50].

Monitoring activities in Puno revealed very similar problems to those in Guatemala. These included illegal charges for services officially covered by the public health insurance program, denials of service, lack of required medicines or equipment, cultural insensitivity, discrimination, and abuse [31, 36, 39, 40, 43]. Many of these problems are tied to systemic weaknesses in the health system that serves rural Indigenous areas. For example, poor families can often be unlawfully charged for certificates of live birth for their new infants, particularly if the delivery did not happen in a health facility [39]. In one study, a nurse explained that her colleagues had collected these kinds of unauthorized charges not for themselves but to pay for informal and unbudgeted costs such as gas for the health centre motorcycle and customary gifts when health workers are asked to take on special roles such as godparents [39]. Many health workers are precariously employed and have little ability to absorb expenses that are not covered by institutional budgets. Other cases, where for example patients are charged for covered medicines, also reveal complex causes. Some of these may be cases of corruption where health workers have direct links to local private pharmacies. In other cases, shortages are the result of problems with staffing and management of supply chains [31, 36, 39]. Additional problems throughout the system arise from the fact that SIS, the public health insurance program, is underfunded. Budget increases have not kept up with the demand for services, nor with the number of services that SIS now covers [51]. In 2019, SIS entitlement was expanded to cover all residents lacking access to another health insurance mechanism [52]. This change resulted in coverage of an additional 1.3 million people without a matching increase in budget. Even before this change, SIS could often be backlogged with debts to hospitals and health networks. Where SIS is slow to send the necessary reimbursement for medicines, this creates further delays in the supply chain [39, 43]. The burden of all of these systemic problems is passed on to health system users, many of whom are members of marginalized communities, especially Indigenous peoples. These dynamics further erode the trust of excluded populations in the county’s publicly-provided system [39].

### Comparing the two cases

Citizen-led accountability initiatives in Guatemala and Peru reveal very similar patterns of serious deficiencies that undermine efforts towards the realization of Universal Health Coverage in both countries. The picture that emerges in each case is of a dysfunctional publicly provided health system marked by gaps and often invisible barriers, particularly for socially excluded groups. The evidence revealed through citizen-led accountability suggests that marginalized health users in Guatemala and Peru do not always receive either the health care services or the protection against financial hardship promised by the publicly provided health systems in each country. These problems are compounded when socially excluded populations are not fully aware of their formal entitlements to a range of services through their public health facilities. Health coverage guaranteed by the state may exist on paper in a way it does not in reality. While many government services can exhibit a gap between promise and reality, the experiences of the citizen-led accountability initiatives reviewed in this paper suggest that the health systems serving rural Indigenous peoples and other socially excluded populations face unusually serious and systemic problems with not only financing but also management, the quality of service provision, and a lack of effective, democratic mechanisms to strengthen governance and accountability.

## Discussion

A focus on the dynamics of social exclusion suggests a partial explanation. Social exclusion is the outcome of ongoing evolving processes that marginalize certain groups within power relations [15, 18]. Socially excluded groups are given fewer rights and entitlements than other segments of society. In addition, their subordinate position in power relations means they are also less able to defend the social rights and entitlements that they do possess. When health systems serve people who lack power, the systems themselves are particularly vulnerable to disregard and abuse. These issues are not often reflected in mainstream policy discussions related to UHC. For example, the official statistics concerning those who spend out of pocket to cover health costs in Guatemala generally reflect the problems of the urban, middle class. What is not captured in official statistics are the frequent payments made by the rural poor for supposedly free services or for the private purchase of essential medical supplies and drugs unavailable in their public health facilities. As a result, the problems faced by the rural poor are not reflected in discussions over out-of-pocket expenses (Walter Flores, August 30, 2019, oral communication with co-authors; unreferenced).

Pressing for accountability from public service providers is intensely difficult, particularly for socially excluded populations. This can translate into impunity for those who might abuse these systems. Also, despite official discourses of citizenship and equality, powerful ideologies circulate in which socially excluded people are defined as undeserving, and are often cast as the recipients of charity rather than as people entitled to health and other social rights [39, 53]. As a result, health systems designed to serve socially excluded groups are liable to be underfunded and mismanaged, to tolerate abusive behaviour, or to have their over-stretched staff deny service as a strategy to manage their own precarious working conditions. Furthermore, we lack mechanisms for identifying and addressing these problems. National indicators for UHC do not reflect the realities faced by the most marginalized populations within highly unequal countries like Guatemala and Peru. The SDGs also provide only limited support towards promoting government accountability in health systems. Although accountability goals exist and procedures for them are recommended within the SDGs, the framework has been justly critiqued for relying on weak voluntary measures rather than institutionalized, obligatory accountability measures [54].

It is telling that these equity and quality issues are evident in Peru, where free publicly provided health coverage for the poorest segments of society has officially expanded over the last decade. This increase in coverage represents a shift towards greater inclusion by expanding certain social rights. However, it is partly an expansion of discursive, not necessarily real access to health services. This shift does not adequately alter the dysfunctional character of the system. Some social rights to coverage are expanded but the quality, accessibility and reliability of that coverage continues to be compromised. This suggests that there may be limits to what can be achieved by pursuing UHC without challenging “social segregation” in health care [21]. Improving those segments of the health system that serve marginalized groups is a positive step, however it also fails to challenge a social divide that normalizes unequal treatment and undermines the rights provided to marginalized groups.

The challenges to realizing UHC in health care systems in Guatemala and Peru are complex. We argue that progress towards ‘democratizing the state’, including the strengthening of democratic mechanisms within public institutions, is crucial. Important steps on this path were taken throughout Latin America in the 1990s, when many countries transitioned from authoritarian regimes [55]. However, a deeper struggle continues to be waged that seeks to counter racialized, ethnic and other hierarchies that were formed long ago in the colonial period and still persist in updated forms in both the state and society [56]. Democratization in this sense involves promoting civic participation, social inclusion and extending real rights to the most marginalized. Grassroots accountability initiatives are part of a broader movement of efforts to improve democratic practices by encouraging marginalized people to make rights claims and by making states more responsive. These kinds of efforts entail substantial challenges when moving from civil society engagement to actions that lead to substantive changes in publicly provided health systems. Experiences such as the National Health Assembly (NHA) model in Thailand are instructive. Development of the NHA model has greatly expanded public participation in health policy making in the country. However, despite these advances in public engagement, researchers report persistent difficulties with convincing key health system officials to effectively implement the NHA resolutions [57].

## Conclusion

Both Guatemala and Peru are middle income countries that have faced challenges in consolidating democratic practices even as their economies have grown. In both countries, social exclusion remains embedded in the state and undermines the health coverage provided to marginalized populations, as well as equitable progress towards UHC. Many other countries face similar predicaments. There is a great deal that donors, governments, and other actors can do to promote UHC for socially excluded populations by helping to democratize and strengthen health systems. Civil society actors and pro-accountability actors within the state need support and institutionalized mechanisms to help them to leverage change. This could include developing specialized participatory spaces for policy dialogue and accountability processes within health systems. Decisions made in these spaces need to be given some binding effect: research suggests that such mechanisms need to provide both “voice” and “teeth” to be effective [29, 57]. Legal empowerment strategies and effective access to information about health rights and participatory rights for marginalized groups are also highly important to pro-accountability efforts [30]. At present, few UHC donor initiatives or government programs focus on democratizing and promoting effective accountability within health systems for socially excluded health users. Reversing this trend is an important step in promoting equitable progress towards UHC.

## Data Availability

Not applicable.
